# Calendar

**DOI:** 10.1155/S1463924682000376

**Published:** 1982


### Calendar

### Editor's note

*Organizers of conferences, seminars, etc. should send details for inclusion in the calendar as soon as the relevant information is available and not later than three months before the event.*

### July 1982

**Swansea Summer School of Automatic Chemical Analysis**
To be held from 11–16 July at University College, Swansea, UK.
*Contact Professor D. Betteridge, Chemistry Department, University College, Singleton Park, Swansea SA2 8PP, UK.*

### August 1982

**9th International Mass Spectrometry Conference**
To be held from 30 August to 3 September in Vienna.
*Contact Interconvention, PO Box 80, A 1107 Vienna.*

### September 1982

**14th Japanese Congress on Clinical Laboratory Automation**
Organized by the Japanese Society of Clinical Laboratory Automation. To be held from 10 to 11 September in Kobe, Japan.
*Contact Dr Koki Motege, c/o Tokyo Clinical Laboratory, 9–3 Nakamarucho, Habashi-ku, Tokyo.*

**Safety of Electrical Instrumentation in Potentially Explosive Atmospheres**
A three-day residential course at UMIST, Manchester, UK to be held from 14–16 September. The course will be repeated at Cudham Hall, Cudham, Kent from 23–25 November.
*Contact the Conference Unit, Sira Institute Ltd, South Hill, Chislehurst, Kent BR7 5EH, UK. Tel.: 01 467 2636.*

**Microcomputers in Chemical Instrumentation**
Intensive five-day course organized by the Chemistry Microprocessor Unit of the Chemistry Department at Imperial College, London. To be held from 20 to 24 September at Imperial College.
*Contact Dr Nick Goddard, Chemistry Microprocessor Unit, Department of Chemistry, Imperial College, London SW7 2AY.*

**Fifth International Symposium on Analytical Pyrolysis**
Supported by the Colorado School of Mines and the University of Utah. To be held from 26–30 September at the Lodge of Vail in Colorado, USA.
*Contact Annette Reed, Office of Special Programs/Continuing Education, Colorado School of Mines, Golden, Colorado 80401, USA.*

**Computer Systems for Safety and Control**
Course to be held from 22–23 September in London.
*Contact Leslie Claff, Oyez IBC Ltd, Norwich House, 11–13 Norwich Street, London EC4A 1AB.*

**Automatic Sampling, Sample Preparation and Presentation Symposium**
Organized by the North East Region and the Automatic Methods Group of the Royal Society of Chemistry. To be held on 30 September and 1 October at the University of York.
*Contact Dr C. J. Jackson, HSE Labs, 403 Edgware Road, London NW2 6LN.*

### October 1982

**Electrochemical Detection in Flow Analysis**
To be held from 17–20 October in Matrafüred, Hungary.
*Contact the Electroanalytical Committee of the Hungarian Academy of Sciences, Budapest.*

**29th Japanese Congress for Clinical Pathologists**
Organized by the Japanese Society of Clinical Pathologists. To be held from 22 to 24 October in Gifu, Japan.
*Contact Dr Nozomu Kosakai, c/o Juntendo University Medical School, Department of Clinical Pathology, 2–1–1 Hongo, Bunkyo-ku, Tokyo 113.*

### March 1983

**34th Pittsburgh Conference and Exposition on Analytical Chemistry and Applied Spectroscopy**
To be held from 7–12 March in Atlantic City, New Jersey, USA.
*Authors should contact Mrs Linda Briggs, Program Secretary, Pittsburgh Conference, 437 Donald Road, Department J-212, Pittsburgh, Pennsylvania 15235, USA; and exhibitors Mr Paul E. Bauer, Exposition Chairman, at the same address.*

### April 1983

**International Symposium on Electroanalysis in Biomedical, Environmental and Industrial Sciences**
Organized by the Electroanalytical Group and Western Region of the Analytical Division of the Royal Society of Chemistry. To be held from 5–8 April at UWIST, Cardiff, UK.
*Contact the Short Courses Section, UWIST, King Edward VII Avenue, Cardiff CF1 3NU, UK.*

### June 1983

**Modern Methods of Food Analysis**
A basic science symposium organized by the Institute of Foods Technologists. To be held from 17–18 June at New Orleans, USA.
*Contact either Dr John Whitaker, University of California, Davis, California 95616, USA; or Dr Kent K. Stewart, Department of Food Science & Technology, Virginia Polytechnic Institute and State University, Blacksburg, Virginia 24061, USA.*

### July 1983

**SAC 83: International Conference and Exhibition on Analytical Chemistry**
Organized by the Analytical Division of the Royal Society of Chemistry. To be held from 17–23 July at the University of Edinburgh.
*Contact Miss P. E. Hutchinson, Analytical Division, Royal Society of Chemistry, Burlington House, London W1V 0BN.*

**Tectronica 83**
To be held in London in June 1983.
*Contact Industrial & Trade Fairs Ltd, Radcliffe House, Blenheim Court, Solihull, West Midlands B91 2BG, UK.*

### November 1984

**14th Annual Symposium on the Analytical Chemistry of Pollutants and the 3rd International Congress on Analytical Techniques in Environmental Chemistry**
Organized by the International Association of Environmental Analytical Chemistry, the Societat Catalana de Ciències (IEC) and Expoquimia. To be held from 22–24 November 1984 at the Palacio de Congresos in Barcelona, Spain.
*For further information contact 14th Annual Symposium—3rd International Congress—Expoquimia, Avda. Reina M.ª Christina, Barcelona 4, Spain.*

